# Bedside EEG predicts longitudinal behavioural changes in disorders of consciousness

**DOI:** 10.1016/j.nicl.2020.102372

**Published:** 2020-08-05

**Authors:** Corinne A. Bareham, Neil Roberts, Judith Allanson, Peter J.A. Hutchinson, John D. Pickard, David K. Menon, Srivas Chennu

**Affiliations:** a Department of Clinical Neurosciences, University of Cambridge, Cambridge, United Kingdom; b Cambridge University Hospitals NHS Foundation Trust, Cambridge, United Kingdom; c Sawbridgeworth Medical Services, Jacobs & Gardens Neuro Centres, Sawbridgeworth, United Kingdom; d Division of Anaesthesia, University of Cambridge, Cambridge, United Kingdom; e School of Computing, University of Kent, Canterbury, United Kingdom

**Keywords:** EEG, Natural history studies (prognosis), Coma, Prognosis, Disorders of consciousness

## Abstract

Providing an accurate prognosis for prolonged disorder of consciousness (pDOC) patients remains a clinical challenge. Large cross-sectional studies have demonstrated the diagnostic and prognostic value of functional brain networks measured using high-density electroencephalography (hdEEG). Nonetheless, the prognostic value of these neural measures has yet to be assessed by longitudinal follow-up. We address this gap by assessing the utility of hdEEG to prognosticate long-term behavioural outcome, employing longitudinal data collected from a cohort of patients assessed systematically with resting hdEEG and the Coma Recovery Scale-Revised (CRS-R) at the bedside over a period of two years. We used canonical correlation analysis to relate clinical (including CRS-R scores combined with demographic variables) and hdEEG variables to each other. This analysis revealed that the patient’s age, and the hdEEG theta band power and alpha band connectivity, contributed most significantly to the relationship between hdEEG and clinical variables. Further, we found that hdEEG measures recorded at the time of assessment augmented clinical measures in predicting CRS-R scores at the next assessment. Moreover, the rate of hdEEG change not only predicted later changes in CRS-R scores, but also outperformed clinical measures in terms of prognostic power. Together, these findings suggest that improvements in functional brain networks precede changes in behavioural awareness in pDOC. We demonstrate here that bedside hdEEG assessments conducted at specialist nursing homes are feasible, have clinical utility, and can complement clinical knowledge and systematic behavioural assessments to inform prognosis and care.

## Highlights

• pDOC patients are assessed for 2-years longitudinally with both the CRS-R and EEG.
• Theta power and alpha clustering correlated strongest with changes in CRS-R scores.
• EEG combined with CRS-R improved predictive power of future CRS-R scores.
• Early changes in EEG outperformed early changes in CRS-R in terms of prognostic power.
• Regular and repeated bedside EEG is feasible and has clinical utility for pDOC.

## 1 Introduction

### 1.1 The prognostic challenge in pDOC

Clinicians face a difficult challenge at predicting the longer-term outcome of pDOC patients following brain injury. This is in part due to a lack of studies that have tracked patients systematically to characterise the history of recovery. Longitudinal studies that undertake systematic follow-up of such patients are challenging as, after acute care, many patients are transferred to specialist neurological centres, nursing homes or repatriated to the family home, with incomplete records of the clinical course and outcomes. As such, most of the studies of pDOC are cross-sectional with convenience samples of patients that do not inform the journey of recovery. Clinical practice guidelines highlight the need for more longitudinal studies to assist with understanding long-term recovery following severe brain injury (Royal College of Physicians, 2020, Giacino et al., 2018, Royal College of Physicians, 2020).

Recently, we aimed to address this need by conducting a longitudinal assessment of 39 pDOC patients to describe the long-term trajectory of behavioural awareness (Bareham et al., 2019). Patients were assessed at the bedside with the Coma Recovery Scale-Revised (CRS-R) once every 3 months for a period of 2 years, allowing for the assessment of longer-term outcomes. The analysis showed statistically significant recovery in behaviour recorded by the CRS-R, with improvements observed beyond 12 months post injury - supporting the proposal to abandon diagnoses of permanence (Giacino et al., 2018). This finding highlighted that many patients may show evidence of late behavioural improvements, a pattern in the natural history of recovery that can be detected with systematic longitudinal follow-up. Also in line with the update to United States (U.S.) practice guidelines (Giacino et al., 2018), the study identified that patients’ age, time since injury and initial CRS-R diagnosis were important predictors of long-term recovery, highlighting these factors as important for prognostication. The study also formally assessed, for the first time, the effect of arousal on CRS-R trajectories (Bareham et al., 2019). Arousal had a significant effect on the behavioural assessment of awareness and was a significant predictor of behavioural trajectories.

### 1.2 Neuroimaging methods to assist prognostication

Recent advances in neuroimaging have indicated that certain neural correlates are associated with the state of consciousness. In particular, research with functional Magnetic Resonance Imaging (fMRI) has indicated that default mode network activity is associated with conscious state in pDOC (Vanhaudenhuyse et al., 2010, Fernández-Espejo et al., 2012, Demertzi et al., 2015). fMRI has also been the prominent approach to detect covert awareness in a minority of patients (Owen et al., 2006, Naci et al., 2014). Problematically, fMRI is unlikely to be viable as a neuroimaging tool for diagnosis and prognosis as it is often not available, feasible for the patient, or affordable. Without regular follow ups to capture variable and delayed changes in behavioural awareness, the prognostic value of fMRI assessments for pDOC is difficult to determine.

High density electroencephalography (hdEEG) is a technique that can be used for regular and repeated assessment at the patient’s bedside. Resting state functional brain networks measured with hdEEG have been shown to be associated with behavioural state in pDOC (Sitt et al., 2014, King et al., 2013, Chennu et al., 2017, Chennu et al., 2014). In particular, structured networks of alpha band connectivity have been shown to reflect the level of behavioural awareness in both patients (Chennu et al., 2017, Chennu et al., 2014) and in healthy people transitioning in and out of sedation (Chennu et al., 2016). This hdEEG indicator of consciousness has also been shown to have prognostic value (Chennu et al., 2017). This research motivated the prospective BETADOC (Bedside Test of Awareness in Disorders of Conscious) study, to demonstrate the prognostic utility of this hdEEG measure by longitudinally monitoring individual patients at the bedside to determine whether accurate estimates of brain network activity can predict behavioural changes. Using case studies from the BETADOC study, we have already demonstrated that hdEEG measures can capture the stability and recovery of behavioural awareness over time (Bareham et al., 2018). Moreover, the hdEEG measures shown to best discriminate between groups of patients (Chennu et al., 2017) were similarly shown to track the progression across conscious states within the context of an individual patient. These promising findings suggest that hdEEG could have clinical value for prognostication.

### 1.3 The BETADOC study

With a novel framework that employs a combination of behavioural and brain-based methods to assess patients’ consciousness state, here we report findings from one of the first longitudinal multimodal studies that systematically follows up a group of pDOC patients. Patients were assessed at the bedside using both the CRS-R and resting state hdEEG measures once every 3 months for a period of 2 years. In total, we collected and analysed 185 assessments from 40 patients recruited across the course of the study.

Firstly, we conducted Canonical Correlation Analysis (CCA) to relate the hdEEG and clinical measures to each other. This approach allows for the analysis of the association between two sets of data - the hdEEG measures that capture the structure of functional brain networks on the one hand, and clinical (including behavioural and demographic) measures on the other (Bareham et al., 2019). Previous analysis of these clinical measures identified arousal as an important predictor of behavioural recovery (Bareham et al., 2019). Given that previous research has identified that these hdEEG measures are closely linked with consciousness state (Chennu et al., 2017), we predicted that arousal will similarly be an important correlate of the longitudinal trajectory of the hdEEG measures. Secondly, we used the canonical scores from the CCA, which independently summarise the pattern of hdEEG and clinical data over time, to determine whether these measures can be used to prognosticate behavioural changes in awareness.

## 2 Materials and methods

### 2.1 Standard protocol Approvals, Registrations, and patient consents

This study was carried out in accordance with the United Kingdom (UK) National Health Service Research Ethics Committee for Cambridgeshire (reference: 16/EE/0006) recommendations. Patients' consultee, or in the absence of a suitable consultee the ward manager acted as a nominated consultee, provided written informed consent prior to enrolment in accordance with the UK Mental Capacity Act 2005 and Declaration of Helsinki.

### 2.2 Patients description

**Fig. 1:**
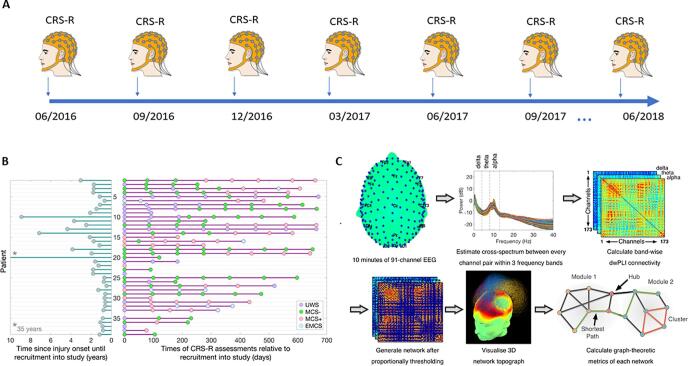
Study design and data processing pipeline. Fig. 1: A. Illustration of the longitudinal design of the project. Patients were assessed at the bedside every 3-months with the CRS-R. Data collection began in June 2016 and was completed in June 2018. Patients were recruited at any point in the data collection period up until February 2018 to obtain a minimum of two assessments. B. Figure illustrating, for each patient, time elapsed since injury onset at the point of recruitment (left), alongside the timeline of individual assessments and CRS-R diagnoses (right). Patients are ordered by time of recruitment into the study, and those recruited later had fewer assessments at the end of the 2-year study period. C. Data Processing Pipeline for Connectivity Analysis - Methodology was identical to (Chennu et al., 2017). Cross-spectral density between pairs of channels was estimated using dwPLI. Resulting connectivity matrices were proportionally thresholded. Thresholded connectivity matrices were visualized as topographs, which combined information about the topography of connectivity with the modular topology of the network. Graph-theoretic metrics were then calculated after binarising the thresholded connectivity matrices.

Patients were recruited from and assessed at two specialist neurological rehabilitation centres, where they received consistent and specialised care throughout the study. For study inclusion, patients needed to be aged 16 years or older and clinically diagnosed as pDOC following any form of sudden onset, non-progressive brain injury. They had to be referred to, or under review of, a consultant in rehabilitation medicine or consultant neurologist. Patients were excluded in the instance of pregnancy, if they were clinically unstable or, if they were diagnosed with a serious mental health condition prior to their brain injury that has required active management by a psychiatrist. Patients who emerged from a DOC immediately prior to recruitment were also excluded. We retained patients who emerged from pDOC during the study, although no further assessments were conducted on these patients after their emergence (see supplementary Table 1). All medications were recorded at the time of recruitment. Forty patients were recruited, however one patient died prior to the first scheduled assessment. The analyses presented here are based on the remaining 39 patients (see Fig. 1B). Of these, 16 had an initial CRS-R diagnosis of UWS, 15 were MCS- (Minimally conscious minus; no evidence of command following) and 7 MCS+ (Minimally conscious plus; evidence of command following) and 1 EMCS (Emerged from a minimally conscious state). MCS- and MCS + patients were categorised based on the level of complexity of observed behaviours, consistent with the definition from Bruno et al. (Bruno et al., 2011). 18 patients had an aetiology of traumatic brain injury (TBI), with the remaining 22 had an anoxic (14), stroke (5) or other (2) injury. The patients (22 Male, 17 Female) were aged 19–75 years (M = 42.85, SD = 15.75) and were 174–12880 days post ictus (M = 1018.64, SD = 2056.77) at the time of the first assessment (see Fig. 1B). Over the course of the 2-year data collection period, 24 patients changed CRS-R diagnosis; 14 progressed from UWS to MCS-/MCS+/EMCS (N = 1) and 10 from MCS-/+ to MCS+/EMCS. The patient that initially had a CRS-R diagnosis of EMCS declined on later assessments to an MCS + diagnosis (see supplementary Table 1). A subset of data from patients 3, 10, 18 and 21 were reported in a previous study (Bareham et al., 2018).

### 2.3 Design

The same researcher (CAB) assessed the patients once every 3 months at the bedside using the CRS-R to determine changes in behaviour as well as hdEEG at rest (see Fig. 1A). The CRS-R was conducted prior to the hdEEG recording to ensure hdEEG was recorded at patient’s peak arousal.

### 2.4 Data availability statement

In total, data from 183 assessments from 39 patients were included in the analysis and has been made available in the supplementary material.

Fig. 1

### 2.5 Coma recovery Scale-Revised

The CRS-R is a 23-item scale behavioural assessment of awareness for pDOC (Giacino et al., 2004). The scale is split into auditory, visual, motor, oromotor/verbal, communication, and arousal subscales. Where possible, the patient was assessed upright in the chair. If this was not possible, patients were assessed at the bedside with the bed elevated to an upright sitting position. When it was required, the arousal intervention of applying deep pressure as per the CRS-R guidelines was administered prior to and, if necessary, throughout the duration of the examination to ensure the patient maintained peak possible arousal.

### 2.6 High-density EEG resting state

Fifteen minutes of resting state data was collected using a 128-channel saline electrode net (Phillips Neuro/Electric Geodesics Inc.) Data were collected at a sampling rate of 500 Hz and were later down-sampled to 250 Hz offline. Prior to EEG collection, the CRS-R was administered to assist with ensuring patients were awake with their eyes open. Patients' behaviours and EEG data were monitored online to ensure recordings were free from seizure activity.

The EEG pre-processing and artifact rejection method was identical to (Chennu et al., 2017) (see supplementary methods). An average of 15 electrodes were rejected and interpolated (range 1–43). The number of rejected trials range from 1 to 53 (Mean = 17). ICA was used to remove muscle artefacts, eyeblinks and in some occasions heartbeat artefacts. On average, 30 such noisy ICA components were removed (range 2–53). The data analysis pipeline was identical (Chennu et al., 2017) (see supplementary methods). Briefly, the debiased weighted phase lag index (dwPLI) (Vinck et al., 2011) metric of connectivity was estimated and graph theory metrics were computed to describe the pattern of this connectivity between electrodes. These measures and the 3D network topography (see Fig. 1C) were visualised alongside those from controls and other patient groups in a feedback reports given to family, in addition to being entered to statistical models. The data analysis pipeline was implemented using EEGLAB (Delorme and Makeig, 2004), Fieldtrip (Oostenveld et al., 2011), the Brain Connectivity Toolbox (Rubinov and Sporns, 2010), and custom MATLAB scripts. The pipeline was automated except for manual checks for and removal of artifactual channels, trials and independent components.

### 2.7 Canonical correlation analysis

Canonical correlation analysis (CCA) seeks maximal correlations between two sets of data. Drawing on the approach used by (Smith et al., 2015), we were interested in the correlation between the hdEEG variables on the one hand, and the clinical variables on the other. The 13 clinical variables were Age, Aetiology, Days since onset of injury, Gender, Initial Diagnosis (CRS-R diagnosis at the first assessment), Patient Number and Assessment Number, as well as each of the scores on the CRS-R subscales.

The hdEEG variables included the mean and standard deviation of the spectral power, as well as the median and standard deviation (over the 91 electrodes) of the dwPLI connectivity in the delta, theta and alpha frequency bands. Additionally, we computed graph-theoretic variables summarising the connectivity “network”, by thresholding, binarising and then modelling the 91 × 91 connectivity matrix as graphs. These graph measures were estimated at each connection density threshold between 0.1 and 0.9, in steps of 0.025. Briefly, the clustering coefficient of a network captures its local efficiency (Watts and Strogatz, 1998), while the characteristic path length measures the average topological distance between pairs of nodes in a graph, providing a measure of global efficiency (Watts and Strogatz, 1998). Modularity, calculated here using the Louvain algorithm (Blondel et al., 2008), is a network metric that captures the degree to which the nodes of a network can be parcellated into densely connected, topologically distinct modules (Fortunato, 2010). Given a modular decomposition, the participation coefficient of a node is an inter-modular measure of its centrality. Finally, modular span is average weighted topographical distance (over the scalp) spanned by a module identified in a network (Chennu et al., 2014). To combine the graph theory values across multiple thresholds before entering them into the CCA, Principal component analysis (PCA) was run over band-wise values at computed thresholds, and the score of the first component was included, which explained an average of 72% (SD = 8.7%) of the variance.

In total, we included 42 variables derived from hdEEG, 14 in each of the 3 frequency bands (see supplementary Table 2 for a full list of hdEEG variables).

The clinical (N = 13) and EEG (N = 42) datasets were submitted to CCA using the *canoncorr* function in MATLAB. The CCA was run with significance testing estimated non-parametrically, using 2000 randomised permutations of the rows of the behavioural and demographic variables relative to the hdEEG variables. These permutations, while destroying the statistical relationship between the variables, respected the repeated-measures structure of the data and shuffled the order of the patients while preserving all the assessments from each patient in the same relative order. The resulting CCA produces a set of canonical variates that captures the linear combination of variables that produces the strongest correlation between the two datasets. Each variate has a score for each patient’s assessment (183 scores per variate).

### 2.8 Linear Mixed Effects model

To investigative the potential additive prognostic value of the hdEEG to the clinical measures, we fitted a Linear Mixed Effects Model (LMEM). Values from the EEG and clinical canonical variates (calculated by the CCA above) from the previous assessment were entered as predictors of the current CRS-R score. As described above, the canonical variate is effectively a weighted mixture of one group of variables that is maximally correlated with a weighted mixture of another group of variables. We entered the canonical variate in the LMEM, instead of the EEG measures themselves, to reduce the dimensionality of the model and thereby increase sensitivity of the LMEM analysis.

Only patients with at least two clinical and hdEEG assessments conducted at two separate time points could be included in this analysis (N = 36 patients, 144 data points). The model included a random factor of patient number to account for the different number of observations per patient. The model also included the intercept of time since the first assessment (assessment number) to account for the longitudinal nature of the data. The dependent variable of CRS-R score was normalised using a Gaussian rank inverse normalisation method (Waerden, 1952) to achieve a Gaussian distribution of values and avoid any influence of potential outliers. The predictors of clinical and hdEEG canonical variate (CV) scores from the previous assessment were also normalised to avoid any influence of outliers. The analysis was conducted using syntax in SPSS software, where the LMEM model equation was as follows:

$$\text{y}=\text{intercept}\left(\text{assessment}\;\text{number}\right)+\text{random}\;\text{factor}\left(\text{patient}\;\text{number}\right)+\text{fixed}\;\text{factors}\left(\text{clinical}\;\text{variate},\;\text{hdEEG}\;\text{variate}\right)$$

### 2.9 Linear stepwise regression

To understand how the rate of change in the hdEEG measures might predict future change in CRS-R scores, we conducted a linear stepwise regression on the data from patients that had at least 4 assessments (N = 23 patients, 46 difference scores). The choice of 4 completed assessments was motivated by the fact that including patients with 5 or more assessments reduced the number of patients and statistical power to a degree that did not allow for formal analysis. We took the rate of change (Δ) of both the hdEEG and the clinical CV values from Assessments 1 to 2 and entered these as predictors of the change in normalised CRS-R scores from Assessments 3 to 4. Using a backward stepwise methodology, predictors were removed sequentially to generate the final model that best explained future CRS-R changes.

## 3 Results

### 3.1 Correlating hdEEG with clinical progression

**Fig. 2:**
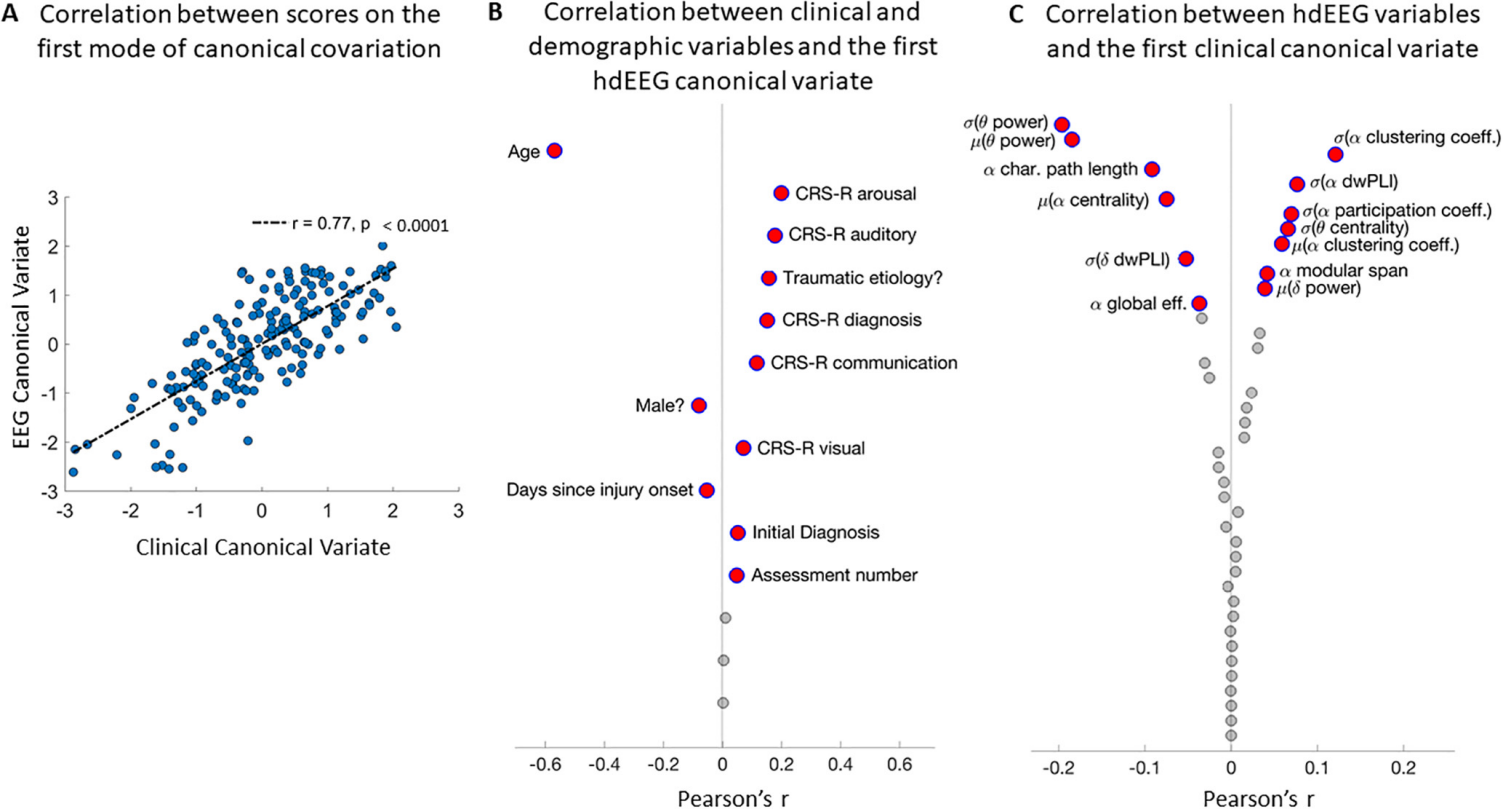
The association between EEG and behaviour over time. Fig. 2: A. Illustration of the correlation between the clinical and EEG variates on the first mode of variation. Each pair of variates at an assessment is plotted as a blue circle. B. Figure illustrating the correlations between the individual clinical variables and the first EEG canonical variate. The clinical variables are ordered by the strength of the correlation, from the strongest (top) to the weakest (bottom). Statistically significant correlations are named and indicated in red. C. Figure illustrating the correlations between the individual EEG variables and the first clinical canonical variate. The EEG variables are ordered by the strength of the correlation, from the strongest (top) to the weakest (bottom). Significant correlations are represented by the variables in red coloured text. (For interpretation of the references to colour in this figure legend, the reader is referred to the web version of this article.)

CCA was used to investigate the relationship between the EEG and clinical measures. The first three pairs of canonical variates (CV) were significantly correlated. However, the first canonical correlation *r* = 0.77, *p* = <0.001 was the strongest and explained the most variance R (Giacino et al., 2018) = 0.033 (see Fig. 2A).

Pearson’s correlations indicated that age was the best predictor of the EEG CV, followed by Arousal (See Fig. 2B). Notably, age was significantly *negatively* associated with the EEG canonical scores indicating that high EEG scores were more likely associated with younger patients. Arousal on the other hand was significantly *positively* correlated indicating that higher EEG scores was associated with higher arousal scores on the CRS-R. These findings are in line with the U.S. clinical practice guidelines (Giacino et al., 2018) and recent findings that age and arousal subscores were important predictors of CRS-R outcome (Bareham et al., 2019).

The strongest correlations between the EEG variables and the first clinical CV were negative correlations with mean and standard deviations of theta power and a positive correlation with clustering and mean dwPLI connectivity in alpha (see Fig. 2C). This indicates that reductions in theta power and increases in alpha connectivity best predicted changes in CRS-R scores over time. This is in line with Bareham et al. (2018) that demonstrated that dwPLI participation coefficient in the alpha band tracked improvements in CRS-R for a patient that progressed from a UWS to MCS- CRS-R diagnosis (Bareham et al., 2018).

### 3.2 Predicting clinical progression using hdEEG

To understand whether hdEEG had any additive value over and above the clinical measures at predicting future CRS-R score, we fitted a Linear Mixed Effects Model (LMEM) to the normalised CRS-R scores predicted by the clinical and hdEEG CVs from the previous assessment. This approach enabled us to enter our data into a single statistical correlation model, without having to resort to pairwise comparisons and consequent correction for multiple comparisons.

**Fig. 3:**
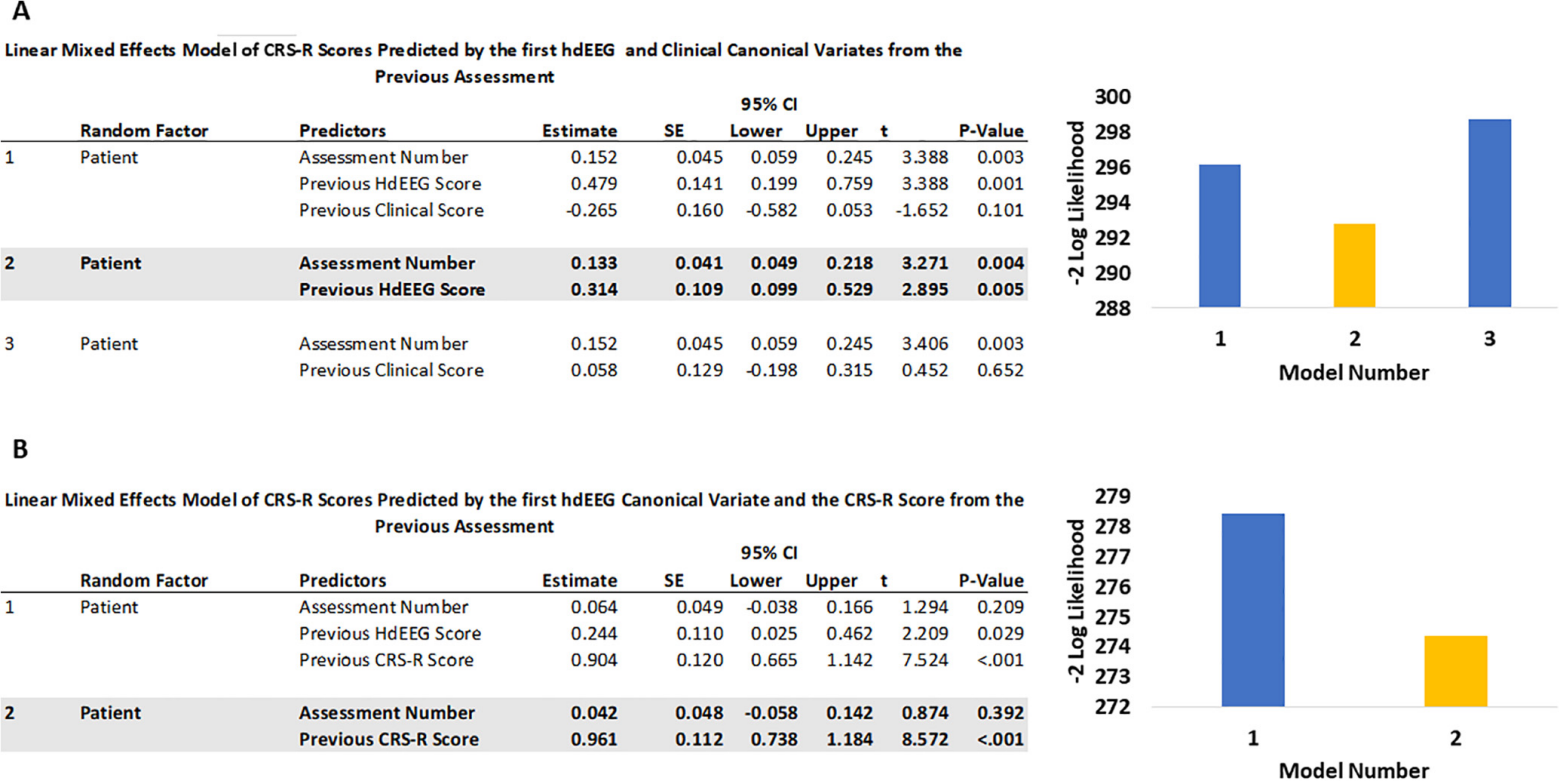
The value of EEG to predict future CRS-R scores. Fig. 3. The Linear Mixed Effects Models of the CRS-R score predicted by the scores on the EEG and clinical canonical variates from the previous assessment using backwards stepwise methodology. Non-significant predictors are removed one by one from the model. The shaded model was the winner, in which a patient’s current CRS-R was predicted only by the previous value of their hdEEG canonical variate. A bar chart of the −2 Log likelihood values is presented for each model. The yellow shaded model 2 had the lowest log likelihood, indicating that it had the relatively lowest amount of unexplained variance. B. The Linear Mixed Effects Models of the patient’s current CRS-R score predicted by the hdEEG canonical variate and CRS-R score from the previous assessment using backwards stepwise methodology. The winning model only included the patient’s previous CRS-R score to predict the current one. A bar chart is presented of the −2 Log likelihood values for each model. (For interpretation of the references to colour in this figure legend, the reader is referred to the web version of this article.)

The LMEM indicated that the assessment number (time) and the EEG CV from the previous assessment were significant predictors of CRS-R score (see Fig. 3A, Model 1). However, the clinical CV from the previous assessment was not a significant predictor. We removed this non-significant predictor and refitted the model (see Fig. 3A, Model 2) which improved the fit, evidenced by the reduced −2 log likelihood (see Fig. 3A) indicating the reduced amount of variance left unexplained. For completeness, we fitted Model 3 to evaluate the predictive power of the clinical CV alone on future CRS-R scores. The increased −2 Log likelihood confirmed (see Fig. 3A) that removal of the hdEEG CV reduced predictive power and led to a poorer model fit.

To determine whether the poor predictive power of the clinical CV was due to its composition of demographic variables with CRS-R subscores, we refitted the LMEM, but this time replacing the clinical CV with normalised CRS-R scores from the previous assessment instead (See Fig. 3B). With this model, the hdEEG CV did not explain any variance over and above the CRS-R score from the previous assessment. In this case, the best-fitting model (Model 2) included only the Assessment Number and the previous CRS-R score, confirmed by comparison of the −2 Log likelihoods (see Fig. 3B), with the variance left unexplained reduced by removal of the hdEEG CV.

### 3.3 Predicting future changes in CRS-R

A limitation of the modelling above was that predicting the subsequent CRS-R score with the previous CRS-R score may lead to an improved model fit from identical scaling. As such, the hdEEG CV may be a poorer predictor simply because the EEG metrics vary on a different scale. To address this, we conducted a subsequent analysis to investigate the prognostic value of the rate of change (Δ) of the hdEEG CV and CRS-R scores on future CRS-R changes. By calculating the rate of change, we put the EEG and normalised CRS-R onto a similar scale, allowing for a fairer juxtaposition of EEG and clinical measures. Moreover, this approach investigated whether early changes in hdEEG predicts later changes in CRS-R scores.

**Fig. 4:**
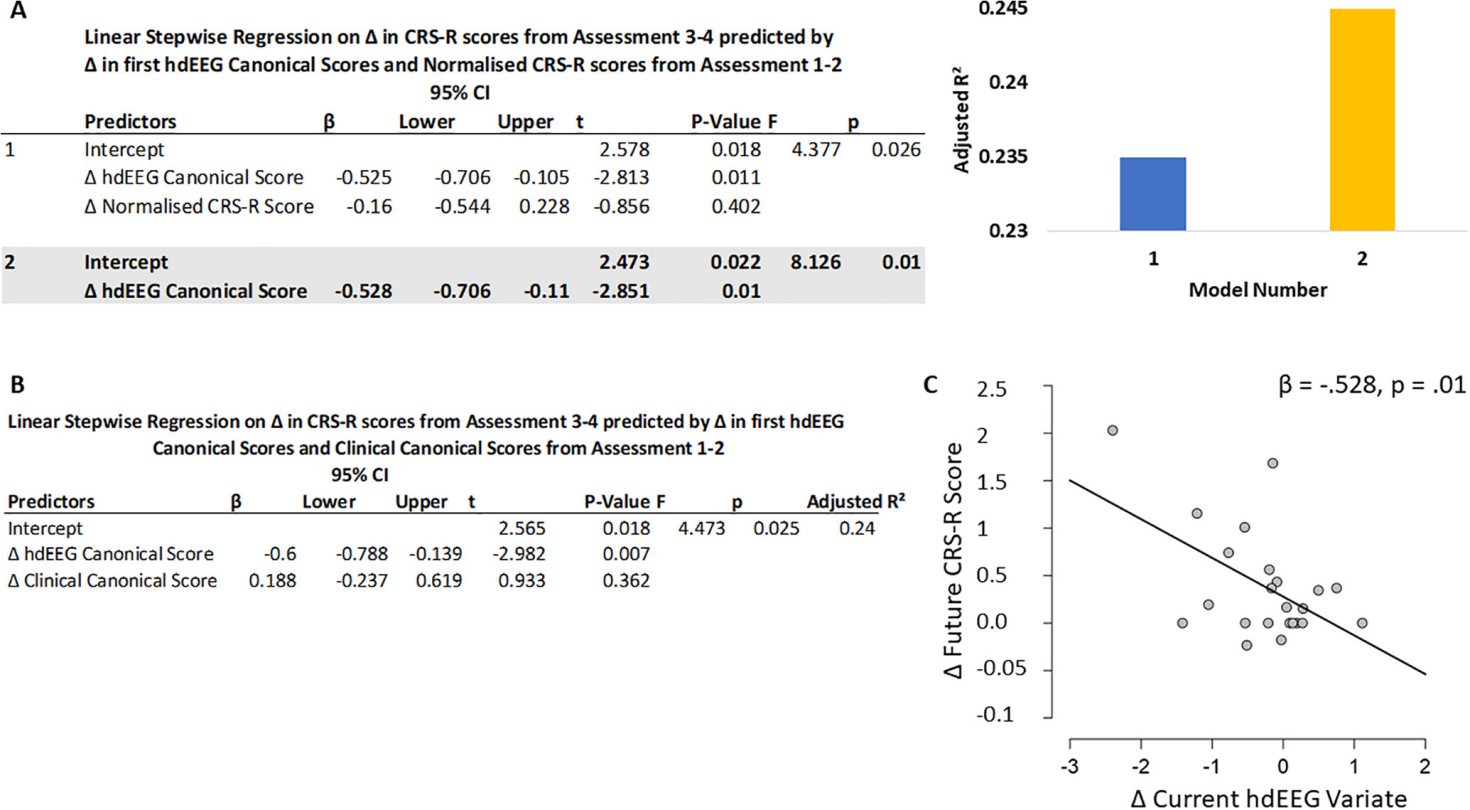
Changes in EEG measures precede changes in CRS-R scores. Fig. 4. A. Linear Stepwise regression of the change in a patient’s CRS-R scores from assessment 3 to 4 predicted by the change in their hdEEG canonical variate and normalised CRS-R scores in assessments 1 to 2. Models are ordered using backwards stepwise methodology with non-significant predictors removed sequentially. The winning model, shaded grey, included only the hdEEG variates as predictors. A bar chart of adjusted R2 values for each model is presented, with the winning model with largest R^2^ highlighted in yellow. B. Linear stepwise regression of the change in CRS-R scores from assessment 3 to 4 predicted by the change in hdEEG and clinical canonical variates from assessment 1 to 2. Comparison of this model to the winning model in panel A shows that this model has less predictive power than the model that includes only the hdEEG canonical variate alone. C. Figure illustrating the linear relationship between the change in CRS-R scores from assessment 3 to 4 and the change in hdEEG canonical variates from assessment 1 to 2, captured by the winning model in panel A. Each grey circle is a single patient (N = 23). (For interpretation of the references to colour in this figure legend, the reader is referred to the web version of this article.)

Fig. 4A presents the results of the backwards stepwise linear regression. The final winning model removed the CRS-R (note the non-significant t-value of -.856p = 0.402) and instead selected Δ hdEEG CV from Assessment 1 to 2 as the best predictor of Δ CRS-R from Assessment 3 to 4. The predictive power of this winning model is reflected with the increased amount of variance explained (Adjusted R^2^) in comparison to the model including the Δ Normalised CRS-R score predictor (see Fig. 4A).

Finally, we found that the Δ hdEEG CV was also a better predictor than Δ clinical CV (see Fig. 4B; note the t value of −2.982, p = 0.007 for the Δ hdEEG CV whilst Δ clinical CV had a t of only 0.933 and non-significant p value = 0.362). The linear fit of the Δ hdEEG CV to the Δ future CRS-R is visualised in Fig. 4C. Whilst the figure demonstrates a negative correlation between the Δ hdEEG CV and Δ future CRS-R, likely driven by reductions in theta power (the strongest variable that correlated with the hdEEG CV), the strength of the correlation is the most important feature.

### 3.4 Discussion

The BETADOC study has taken a longitudinal approach to assess pDOC patients at the bedside in their resident neurological centre using both behavioural and brain-based methods. This novel framework has allowed for regular, systematic follow-ups with pDOC patients to assess the value of both hdEEG and the CRS-R at predicting long-term outcomes. Our analysis has indicated that the hdEEG measures of theta power and the connectivity measures of clustering and median dwPLI in the alpha band are the strongest contributors to the canonical scores of clinical-behavioural trajectories. Also, patients’ age and arousal levels, already identified in our previous analysis (Bareham et al., 2019) as important predictors of behavioural recovery, were also the strongest correlates of hdEEG canonical variates. In addition, our findings show that hdEEG measures, when combined with clinical measures, improved our ability to predict consequent CRS-R scores. Moreover, our findings show that the rate of hdEEG changes early in the trajectory is the best predictor of later behavioural changes, outperforming the predictive power of early behavioural changes. These findings indicate that resting state hdEEG measures have significant prognostic value for predicting long-term behavioural outcomes in pDOC.

### 3.5 The link between brain and behaviour in pDOC recovery

We took a statistically valid approach (CCA) to assess the relationship between patterns of hdEEG and clinical measures over time. This enabled us to understand how brain and behaviour related to each other in this complex and variable clinical cohort. In doing so, this study extends beyond most of previous research that has pinpointed neural measures most strongly associated with consciousness state usually measured once in a patient, using a cross-sectional design (Vanhaudenhuyse et al., 2010, Sitt et al., 2014, King et al., 2013). Here, over 183 assessments collected systematically from 39 patients every 3 months, we have identified that reductions in theta power and increases in alpha clustering and connectivity are the most important hdEEG measures that correlate with patients’ clinical profiles. This finding is consistent with cross-sectional studies showing that reduced theta power (Chennu et al., 2014) and increases in alpha band connectivity is associated with consciousness state across patient groups (Sitt et al., 2014, Chennu et al., 2017) and with recovery of individual cases (Bareham et al., 2018). Therefore, reduced theta band activity and increased alpha band network connectivity are indicators of higher consciousness levels, or recovery, in individual patients over time. This finding has important clinical implications for the development of a brain-based tool for diagnosis and prognosis. Targeting theta power and alpha connectivity could improve the sensitivity and accuracy of methods that aim to measure consciousness and the potential for recovery.

The CCA approach also allowed us to assess the clinical measures most strongly related with hdEEG network changes over time. We found that along with patients’ age, arousal was an important correlate of hdEEG. We have previously reported that age and arousal are important correlates of behavioural recovery (Bareham et al., 2019). While the role of arousal is clinically known to be important (Giacino et al., 2004, Wilson et al., 1996, Wannez et al., 2017, Seel et al., 2010), this is the first time that arousal has been formally tested as an important correlate of brain-based measures of consciousness.

### 3.6 Arousal is an important predictor of hdEEG trajectories

Alongside the behavioural assessment of consciousness (Giacino et al., 2018, Giacino et al., 2004), an intervention may be required to ensure peak arousal before conducting neuroimaging assessments or when using Brain Computer Interfaces. Also, the influence of arousal is an important consideration in the development of neuroimaging methods for diagnosis and prognosis in this patient cohort. Research into computational methods that aim to produce a valid neural index of consciousness need to appropriately account for fluctuations in arousal that mediate other neural correlates of consciousness such as connectivity and complexity.

Consciousness involves an interaction between two theoretical concepts of arousal and awareness (Laureys, 2005). Whilst arousal is thought to capture the *level* of consciousness, awareness is considered to capture conscious *content*. Our findings demonstrated that changes in arousal correlated with changes in hdEEG measures over time. This finding agrees with the notion that emergence from DOC involves recovery of arousal systems that support brain networks that underpin conscious awareness, perhaps revealing the course of neural mechanisms in the natural history of recovery following brain injury. This proposal is in line with the *meso*-circuit hypothesis (Schiff, 2010), and could explain the beneficial effects of interventions such as amantadine and zolpidem (Giacino et al., 2012, Whyte and Myers, 2009, Whyte et al., 2014), and deep brain stimulation (Schiff et al., 2007), that target regions of the brainstem and thalamus involved in arousal modulation leading to increases in large scale cortical projections that support conscious awareness.

### 3.7 The value of bedside hdEEG to prognosticate behavioural recovery

We directly assessed the prognostic value of the hdEEG measures combined with some of the clinical measures typically used in current settings to assist with clinical decisions of prognosis and care plans. The central finding from our LMEM was that the addition of hdEEG to these clinical measures improved our ability to predict the CRS-R score in the next assessment. This finding implicates that hdEEG adds significant value to prognostication when combined with clinical measures. The current CRS-R score itself was of course a very good predictor of the CRS-R score on a subsequent assessment. But this is not entirely surprising given that, in this case, the independent (predictor) variable and dependent variable are measured on the same scale, whereas the hdEEG canonical scores have undergone a scale transformation.

To determine whether hdEEG is a valuable inclusion to patient assessments we investigated whether early hdEEG changes predict later changes in CRS-R scores. This analysis avoided the scaling issue by keeping all the variables on comparable scales. Our results indicated that not only did change in the hdEEG CV predict later CRS-R changes, hdEEG outperformed the prognostic ability of earlier changes in CRS-R scores. The implication of this finding is that hdEEG would not only be a valuable contribution to routine clinical care to track changes in awareness and assist with diagnosis, it would arm clinicians with stronger evidence to provide accurate prognoses and inform rehabilitation and care plans.

More generally, this finding also indicates that changes in brain network measures captured with hdEEG *precede* changes in behaviour. This finding has been alluded to in previous cross-sectional studies using hdEEG (Sitt et al., 2014, Chennu et al., 2017) but not formally assessed in a longitudinal study until now. Information regarding changes in brain network connectivity may provide more fine-grained information regarding the potential for recovery that presages the behavioural manifestation of consciousness. As such, hdEEG assessments are likely to be more valuable than behavioural measures of awareness to predict behavioural outcomes.

### 3.8 Clinical utility of bedside hdEEG

The current UK clinical practice guidelines note that resting state EEG was not considered to have discriminative utility to assist with diagnosis in pDOC (Physicians RCo, 2020). Since these guidelines have been published, EEG measures of resting state networks have been found to capture important information that can discriminate between diagnostic groups (Chennu et al., 2017, Chennu et al., 2014), and these measures have been shown to be robust enough to significantly predict outcome in pDOC (Chennu et al., 2017). Here, we have utilised these resting state hdEEG measures to systematically assess patients at the bedside in their resident nursing home – an approach that did not require relocation of patients to a specialised setting. This approach highlights the feasibility of potentially conducting bedside EEG assessments regularly as part of routine care to allow for long-term systematic follow up and improved detection of late changes in awareness.

We showed a strong correlation between hdEEG and clinical measures of pDOC over time implicating that hdEEG can provide corroborative evidence for improved pDOC diagnosis. This could be particularly useful when behavioural measures are inconsistent due to arousal fluctuations. Future studies could assess whether sensitivity of hdEEG to arousal fluctuations and whether hdEEG variability can predict CRS-R variability, potentially assisting diagnosis in complex cases. Moreover, arousal was found to be a significant predictor of both behavioural trajectories (Bareham et al., 2019) and hdEEG trajectories in these patients. It is possible that fine grained, rich, hdEEG measures of arousal may surpass the CRS-R arousal subscore in terms of prognostic power. This notion is substantiated by the finding that the rate of hdEEG change surpassed changes in CRS-R in terms of predicting later behavioural changes. As such, hdEEG can improve prognostic information to assist clinical decisions and direct treatment care plans and allocation of therapeutic resources. From a practical perspective, high-density EEG saline net takes approximately 10 min to prepare, on par with low-density EEG systems using conventional gel electrodes. Hence, hdEEG systems could be feasible for clinical bedside assessments despite the density of electrodes.

### 3.9 Limitations

Research has indicated that a minimum of 5 CRS-R assessments are required to ensure consistency (Wannez et al., 2017). In this study, patients were assessed only once every 3 months as multiple assessments were not feasible as the scale of this study only allowed for a single examiner. The arousal sub-scale has been included as a variable in the study to account for potential fluctuations and the examiner administered the arousal intervention when considered necessary, as per CRS-R guidelines (Giacino et al., 2004), to ensure peak arousal prior to assessment. Nevertheless, the use of a single examiner could have led to undetected measurement bias. Future larger scale studies should employ multiple examiners to establish inter-rate reliability between examiners on longitudinal assessments.

Here, we have used the gaussian normalised CRS-R score as an indicator of behavioural change. However, increases in CRS-R do not necessarily indicate improved consciousness state (Bodien et al., 2016). This is accounted for in the CCA with CRS-R diagnosis included as a variable in the analysis, but this was not able to be included in LMEM analyses. Nonetheless, the LMEM results still captures whether EEG can predict behavioural changes as measured by CRS-R.

Due to attrition, and because patients were recruited at any time up until 3 months before the end of data collection, there are a different number of assessments for each patient. To control for this potential source of variation, we included Patient Number as a random factor in statistical models. The patients reported here all resided in specialist centres with access to rehabilitation services that other patients may not have access to. To better characterise the role of the rehabilitation context on patient trajectories, further larger studies could involve patients from multiple centres.

### 3.10 Conclusions

The strength of the longitudinal design in the BETADOC study has revealed important predictors of longer-term outcomes in pDOC. Using CCA, we identified the hdEEG measures most strongly linked with behavioural and demographic variables. The prognostic value of the hdEEG measures was evident when combined with clinical measures. Moreover, the utility of early hdEEG changes surpassed early behavioural changes in prognosticating later changes in behaviour. The addition of regular and repeated hdEEG to routine care then, will not just benefit current clinical assessments by providing corroborative evidence. Our findings here indicate that hdEEG assessments can further improve the accuracy of clinical prognostication. Crucially, hdEEG measures captured patients’ potential for recovery with changes in hdEEG preceding behavioural changes. Finally, we note that arousal was also an important correlate of hdEEG measures and, as such, arousal levels should be taken into account during diagnostic and prognostic neuroimaging in pDOC.

## 4 Financial disclosures

The authors declare that the research was conducted in the absence of any commercial or financial relationships that could be construed as a potential conflict of interest.

Study Funded by the Wellcome Trust ISSF [CAB, Ref: ISSF/SCM/48], Evelyn Trust [SC, Ref: 15/07], UK Engineering and Physical Sciences Research Council [SC, Ref: EP/P033199/1], the UK National Institute for Health Research (NIHR) as part of the Acute Brain Injury and Repair Theme of the Cambridge Biomedical Research Centre [PJAH], the NIHR Research Professorship [PJAH], the NIHR Cambridge BRC [PJAH and DKM], the NIHR Brain Injury Healthcare Technology Cooperative [JDP], the NIHR Senior Investigator award [DKM] and the James S. McDonnell Foundation [JDP]. This research was undertaken with the support of the Alan Turing Institute (UK Engineering and Physical Sciences Research Council Grant Number EP/N510129/1).

## CRediT authorship contribution statement

**Corinne A. Bareham:** Conceptualization, Methodology, Software, Formal analysis, Investigation, Data curation, Writing - original draft, Visualization, Supervision, Project administration, Funding acquisition, Writing - review & editing. **Neil Roberts:** Resources, Writing - review & editing. **Judith Allanson:** Conceptualization, Resources, Funding acquisition, Writing - review & editing. **Peter J.A. Hutchinson:** Conceptualization, Funding acquisition, Writing - review & editing. **John D. Pickard:** Conceptualization, Funding acquisition, Writing - review & editing. **David K. Menon:** Conceptualization, Funding acquisition, Writing - review & editing. **Srivas Chennu:** Conceptualization, Software, Visualization, Supervision, Project administration, Funding acquisition, Writing - review & editing.

## Declaration of Competing Interest

The authors declare that they have no known competing financial interests or personal relationships that could have appeared to influence the work reported in this paper.

## Figures and Tables

**Table:** 

Name	Location	Role	Contribution
Corinne A. Bareham, PhD	University of Cambridge, Cambridge	Author	Designed and conceptualised the study. Organised the database. Collected the data. Conducted the data analysis. Wrote the manuscript. Manuscript revision, read and approved final manuscript.
Judith Allanson, BM, BCh, PhD, MRCP	Cambridge University Hospitals NHS Foundation Trust, Cambridge	Author	Designed and conceptualised the study. Conducted detailed Neurological examination of the patients. Manuscript revision, read and approved final manuscript.
Neil Roberts, MB, BS, MRCA	Sawbridgeworth Medical Services, Jacobs & Gardens Neuro Centres, Sawbridgeworth	Author	Contributed clinical information about patients. Read and approved the final manuscript.
Peter J. A. Hutchinson, MBBS, PhD, FMedSci	University of Cambridge, Cambridge	Author	Designed and conceptualised the study. Manuscript revision, read and approved final manuscript.
John D. Pickard, MChir, FRCS, FMedSci	University of Cambridge, Cambridge	Author	Designed and conceptualised the study. Manuscript revision, read and approved final manuscript.
David K. Menon, MD, PhD, FRCP, FRCA, FFICM, FMedSci	University of Cambridge, Cambridge	Author	Designed and conceptualised the study. Manuscript revision, read and approved final manuscript.
Srivas Chennu, PhD	University of Kent, Canterbury	Author	Designed and conceptualised the study. Manuscript revision, read and approved final manuscript.

## References

[b0070] Bareham C, Allanson J, Roberts N, et al. Longitudinal bedside assessments of brain networks in disorders of consciousness: case reports from the field. Frontiers in neurology 2018;9:676. 10.3389/fneur.2018.00676 PMC6110818 30186220

[b0015] Bareham CA, Allanson J, Roberts N, et al. Longitudinal assessments highlight long-term behavioural recovery in disorders of consciousness. Brain Communications 2019. 10.1093/braincomms/fcz017 PMC6924536 31886461

[b0115] Blondel VD, Guillaume J-L, Lambiotte R, Lefebvre E. Fast unfolding of communities in large networks. Journal of Statistical Mechanics: Theory and Experiment 2008;2008:P10008.

[b0175] Bodien Y.G., Carlowicz C.A., Chatelle C., Giacino J.T. (2016). Sensitivity and specificity of the coma recovery scale–revised total score in detection of conscious awareness. Archives of physical medicine and rehabilitation.

[b0075] Bruno M.-A., Vanhaudenhuyse A., Thibaut A., Moonen G., Laureys S. (2011). From unresponsive wakefulness to minimally conscious PLUS and functional locked-in syndromes: recent advances in our understanding of disorders of consciousness. J Neurol.

[b0055] Chennu S, Annen J, Wannez S, et al. Brain networks predict metabolism, diagnosis and prognosis at the bedside in disorders of consciousness. Brain 2017;140:2120-2132. 10.1093/brain/awx163 28666351

[b0060] Chennu S, Finoia P, Kamau E, et al. Spectral signatures of reorganised brain networks in disorders of consciousness. PLOS Computational Biology 2014;10:e1003887. 10.1371/journal.pcbi.1003887 PMC4199497 25329398

[b0065] Chennu S, O’Connor S, Adapa R, Menon DK, Bekinschtein TA. Brain Connectivity Dissociates Responsiveness from Drug Exposure during Propofol-Induced Transitions of Consciousness. PLOS Computational Biology 2016;12:e1004669. 10.1371/journal.pcbi.1004669 PMC4713143 26764466

[b0090] Delorme A., Makeig S. (2004). EEGLAB: an open source toolbox for analysis of single-trial EEG dynamics including independent component analysis. Journal of Neuroscience Methods.

[b0030] Demertzi A., Antonopoulos G., Heine L., Voss H.U., Crone J.S., de Los Angeles C., Bahri M.A., Di Perri C., Vanhaudenhuyse A., Charland-Verville V., Kronbichler M., Trinka E., Phillips C., Gomez F., Tshibanda L., Soddu A., Schiff N.D., Whitfield-Gabrieli S., Laureys S. (2015). Intrinsic functional connectivity differentiates minimally conscious from unresponsive patients. Brain.

[b0025] Fernández-Espejo D., Soddu A., Cruse D., Palacios E.M., Junque C., Vanhaudenhuyse A., Rivas E., Newcombe V., Menon D.K., Pickard J.D., Laureys S., Owen A.M. (2012). A role for the default mode network in the bases of disorders of consciousness. Ann Neurol..

[b0120] Fortunato S. (2010). Community detection in graphs. Physics Reports.

[b0080] Giacino J.T., Kalmar K., Whyte J. (2004). The JFK Coma Recovery Scale-Revised: Measurement characteristics and diagnostic utility1. Archives of physical medicine and rehabilitation.

[b0155] Giacino J.T., Whyte J., Bagiella E., Kalmar K., Childs N., Khademi A., Eifert B., Long D., Katz D.I., Cho S., Yablon S.A., Luther M., Hammond F.M., Nordenbo A., Novak P., Mercer W., Maurer-Karattup P., Sherer M. (2012). Placebo-Controlled Trial of Amantadine for Severe Traumatic Brain Injury. N Engl J Med.

[b0010] Giacino J.T., Katz D.I., Schiff N.D., Whyte J., Ashman E.J., Ashwal S., Barbano R., Hammond F.M., Laureys S., Ling G.S.F., Nakase-Richardson R., Seel R.T., Yablon S., Getchius T.S.D., Gronseth G.S., Armstrong M.J. (2018). Practice Guideline Update Recommendations Summary: Disorders of Consciousness. Archives of Physical Medicine and Rehabilitation.

[b0050] King JR, Sitt JD, Faugeras F, et al. Information sharing in the brain indexes consciousness in noncommunicative patients. Curr Biol 2013;23:1914-1919. 10.1016/j.cub.2013.07.075 PMC5635964 24076243

[b0145] Laureys S. (2005). The neural correlate of (un)awareness: lessons from the vegetative state. Trends in Cognitive Sciences.

[b0040] Naci L., Cusack R., Anello M., Owen A.M. (2014). A common neural code for similar conscious experiences in different individuals. Proc Natl Acad Sci USA.

[b0095] Oostenveld R., Fries P., Maris E., Schoffelen J.-M. (2011). FieldTrip: Open Source Software for Advanced Analysis of MEG, EEG, and Invasive Electrophysiological Data. Computational Intelligence and Neuroscience.

[b0035] Owen AM, Coleman MR, Boly M, Davis MH, Laureys S, Pickard JD. Detecting awareness in the vegetative state. Science 2006;313. 10.1126/science.1130197 16959998

[b0005] Royal College of Physicians, 2020. Prolonged disorders of consciousness following sudden onset brain injury: national clinical guidelines. London.

[b0100] Rubinov M., Sporns O. (2010). Complex network measures of brain connectivity: Uses and interpretations. NeuroImage.

[b0150] Schiff N.D. (2010). Recovery of consciousness after brain injury: a mesocircuit hypothesis. Trends in Neurosciences.

[b0170] Schiff N.D., Giacino J.T., Kalmar K., Victor J.D., Baker K., Gerber M., Fritz B., Eisenberg B., O’Connor J., Kobylarz E.J., Farris S., Machado A., McCagg C., Plum F., Fins J.J., Rezai A.R. (2007). Behavioural improvements with thalamic stimulation after severe traumatic brain injury. Nature.

[b0140] Seel R.T., Sherer M., Whyte J., Katz D.I., Giacino J.T., Rosenbaum A.M., Hammond F.M., Kalmar K., Pape T.-B., Zafonte R., Biester R.C., Kaelin D., Kean J., Zasler N. (2010). Assessment Scales for Disorders of Consciousness: Evidence-Based Recommendations for Clinical Practice and Research. Archives of Physical Medicine and Rehabilitation.

[b0045] Sitt JD, King JR, El Karoui I, et al. Large scale screening of neural signatures of consciousness in patients in a vegetative or minimally conscious state. Brain 2014;137:2258-2270. 10.1093/brain/awu141 PMC4610185 24919971

[b0105] Smith S.M., Nichols T.E., Vidaurre D., Winkler A.M., Behrens T.E.J., Glasser M.F., Ugurbil K., Barch D.M., Van Essen D.C., Miller K.L. (2015). A positive-negative mode of population covariation links brain connectivity, demographics and behavior. Nat Neurosci.

[b0020] Vanhaudenhuyse A, Noirhomme Q, Tshibanda LJ-F, et al. Default network connectivity reflects the level of consciousness in non-communicative brain-damaged patients. Brain 2010;133:161-171. 10.1093/brain/awp313 PMC2801329 20034928

[b0085] Vinck M., Oostenveld R., van Wingerden M., Battaglia F., Pennartz C.M.A. (2011). An improved index of phase-synchronization for electrophysiological data in the presence of volume-conduction, noise and sample-size bias. NeuroImage.

[b0110] Watts D.J., Strogatz S.H. (1998). Collective dynamics of ‘small-world’ networks. Nature.

[b0125] Waerden B.L.V.D. (1952). Order tests for the two-sample problem and their power. Indagationes Mathematicae (Proceedings).

[b0130] Wilson S.L., Brock D., Powell G.E., Thwaites H., Elliott K. (1996). Constructing arousal profiles for vegetative state patients-a preliminary report. Brain Injury.

[b0135] Wannez S., Heine L., Thonnard M., Gosseries O., Laureys S. (2017). The repetition of behavioral assessments in diagnosis of disorders of consciousness: Repeated CRS-R Assessments for Diagnosis in DOC. Ann Neurol..

[b0160] Whyte J., Myers R. (2009). Incidence of Clinically Significant Responses to Zolpidem Among Patients with Disorders of Consciousness: A Preliminary Placebo Controlled Trial. American Journal of Physical Medicine & Rehabilitation.

[b0165] Whyte J, Rajan R, Rosenbaum A, et al. Zolpidem and restoration of consciousness. American journal of physical medicine & rehabilitation 2014;93:101-113. 10.1097/PHM.0000000000000069 24434886

