# Supplementary material for: Bedside EEG predicts longitudinal behavioural changes in disorders of consciousness
Source: Neuroimage Clin. 2020 Aug 5;28:102372. doi: 10.1016/j.nicl.2020.102372 (PMC7426558; doi:10.1016/j.nicl.2020.102372)
Supplement: Supplementary data 3 [file mmc3.docx]

**Supplementary Methods**

*Pre-processing and artifact removal stages*

Data from electrodes near the eyes, face and neck was removed, leaving 91 electrodes for further analysis. Data was filtered at 0.5–45 Hz and then epoched to 10-second epochs. Each epoch thus generated was baseline-corrected relative to the mean voltage over the entire epoch. Data containing excessive eye movement or muscular artifact was rejected by a quasi-automated procedure: abnormally noisy channels and epochs were identified by calculating their normalized variance and then manually rejected or retained by visual inspection. Independent Components Analysis (ICA) based on the Infomax ICA algorithm ^1^ was used to visually identify and reject noisy components. Finally, previously rejected channels were interpolated using spherical spline interpolation, and data were re-referenced to the average of all channels.

*Computation of connectivity measures*

Using a multitaper method with five Slepian tapers ^2^, spectral and cross-spectral decompositions within the canonical delta (0.5–4 Hz), theta (4–8 Hz) and alpha (8–13 Hz) frequency bands were computed at bins of 0.1 Hz. Spectral power values were normalized by dividing the power at each bin by the total power over all three bands and multiplying by 100 ^3^. Alongside, the cross-spectral decomposition was used to estimate the debiased weighted phase lag index (dwPLI) ^4^ metric of connectivity between every pair of electrodes. dwPLI minimizes the effects of volume conduction on the estimation of brain connectivity, and is further minimally biased at small sample sizes ^4^. Within each frequency band, dwPLI values at the peak frequency of the oscillatory signal across all channels were used to represent the connectivity between channel pairs. From each subject's dataset, the dwPLI values across all channel pairs were used to construct symmetric 91 × 91 dwPLI connectivity matrices for the delta, theta and alpha bands.

The dwPLI matrices thus constructed were thresholded proportionally to preserve 90–10% of the largest dwPLI values in steps of 2.5%. Specifically, at the 90% threshold, only the 10% of the weakest network edges were discarded. At the 10% threshold, 90% of the weakest edges were discarded. This lowest threshold of 10% ensured that the average degree was not smaller than 2 log(N), where N is the number of nodes in the network (i.e., N = 91). This in turn guaranteed that the resulting networks could be estimated ^5^. Further, graph connection densities within this range of thresholds have been shown to be sensitive to the estimation of “true” topological structure therein ^6, 7^.

After applying each of these thresholds, matrices were binarised, i.e., non-zero values were set to 1. These matrices were then modelled as networks with channels as nodes and binarised dwPLI values as connections between them. These networks were analysed using graph theory algorithms to calculate a pre-defined set of summary metrics previously evaluated in an independent dataset ^8^ – clustering coefficient, characteristic path length, modularity, participation coefficient and modular span – at each value of the proportional threshold. The clustering coefficient of a network captures its local efficiency ^5^, while the characteristic path length measures the average topological distance between pairs of nodes in a graph, providing a measure of global efficiency ^5^. Modularity, calculated here using the Louvain algorithm ^9^, is a network metric that captures the degree to which the nodes of a network can be parcellated into densely connected, topologically distinct modules . Given a modular decomposition, the participation coefficient of a node is an inter-modular measure ^10^ of its centrality ^11^. A larger standard deviation in participation coefficient of network nodes indicates a diversity of connectivity, and hence the presence of hub nodes that link many modules together in an efficient network. Here, we used the standard deviation of participation coefficients to measure network centrality as the presence of diversely connected nodes with central hubs ^12, 13^. Finally, modular span is average weighted topographical distance (over the scalp) spanned by a module identified in a network ^14^. Network metrics were averaged over all connection densities considered, to reduce them down to scalar values when plotting them alongside CRS-R scores.

**Supplementary References**

1. Loewy DH, Campbell KB, Bastien C. The mismatch negativity to frequency deviant stimuli during natural sleep. Electroencephalography and Clinical Neurophysiology 1996;98:493 - 501.

2. Mitra PP, Pesaran B. Analysis of dynamic brain imaging data. Biophysical journal 1999;76:691-708.

3. Sitt JD, King JR, El Karoui I, et al. Large scale screening of neural signatures of consciousness in patients in a vegetative or minimally conscious state. Brain 2014;137:2258-2270.

4. Vinck M, Oostenveld R, van Wingerden M, Battaglia F, Pennartz CM. An improved index of phase-synchronization for electrophysiological data in the presence of volume-conduction, noise and sample-size bias. Neuroimage 2011;55:1548-1565.

5. Watts DJ, Strogatz SH. Collective dynamics of 'small-world' networks. Nature 1998;393:440-442.

6. Lynall ME, Bassett DS, Kerwin R, et al. Functional connectivity and brain networks in schizophrenia. J Neurosci 2010;30:9477-9487.

7. Achard S, Delon-Martin C, Vértes PE, et al. Hubs of brain functional networks are radically reorganized in comatose patients. Proc Natl Acad Sci U S A 2012;109:20608-20613.

8. Chennu S, Annen J, Wannez S, et al. Brain networks predict metabolism, diagnosis and prognosis at the bedside in disorders of consciousness. Brain 2017;140:2120-2132.

9. Blondel VD, Guillaume J-L, Lambiotte R, Lefebvre E. Fast unfolding of communities in large networks. Journal of Statistical Mechanics: Theory and Experiment 2008;2008:P10008.

10. Fortunato S. Community detection in graphs. Physics Reports 2010;486:75-174.

11. Guimera R, Nunes Amaral LA. Functional cartography of complex metabolic networks. Nature 2005;433:895-900.

12. Van Den Heuvel MP, Sporns O. Rich-club organization of the human connectome. Journal of Neuroscience 2011;31:15775-15786.

13. Bertolero M, Yeo B, D’esposito M. The diverse club. Nature communications 2017;8:1277.

14. Chennu S, Finoia P, Kamau E, et al. Spectral signatures of reorganised brain networks in disorders of consciousness. PLOS Computational Biology 2014;10:e1003887.
